# Is pull-through an acceptable replacement for low anterior resection for rectal cancers in low-income setting? A case-control study^☆^

**DOI:** 10.1016/j.amsu.2021.102608

**Published:** 2021-07-27

**Authors:** Amjad Ghareeb, Ameer Kakaje, Ayham Ghareeb, Fadi Obaid Alahmar

**Affiliations:** a Faculty of Medicine, Damascus University, Damascus, Syria; b University Hospital Geelong, Barwon Health, Victoria, Australia; c Al Assad University Hospital, Damascus University, Damascus, Syria

**Keywords:** Pull-through, Low anterior resection, Rectal cancer, Trans-anal, Low-income, Stapler, APR, Abdominal perineal resection, CRT, Chemoradiotherapy, FU, Fluorouracil, LAR, Low anterior resection, PT, Pull-through, SPSS, Statistical Package for the Social Sciences, STROCSS, Strengthening the reporting of cohort studies in surgery, TME, Total mesorectal excision

## Abstract

**Background:**

Colorectal cancers are the second most common cancers overall and are the third deadliest cancers. Complete resection is the treatment of choice for rectal cancers and chemoradiotherapy (CRT) is strongly recommended in stage 2 and 3. Low anterior resection (LAR) is the most common procedure used, but it requires the use of stapler which might be very expensive as one study estimated the median cost of LAR inpatients to be over 13.000 USD. However, coloanal pull-through (PT) used to be the common procedure before introducing staplers in the twentieth century and can be less expensive, but with higher complication rates.

**Materials and methods:**

This is a retrospective case-control study from patients’ records who underwent either LAR or PT for their rectal cancer in Syria. All patients had either stage 2 or 3 cancer and were treated by the same group of surgeons and received the same adjuvant and neoadjuvant CRT protocol. Patients from both groups had the same prognosis and stages.

**Results:**

This study included 60 participants, of which, 30 had LAR and 30 had PT. They all had successful removal of the cancer and follow-ups were for 1 year after the surgery. There were no significant differences between the two procedures in post-operative leak, urinary retention, stricture, sexual function and recurrence (p > 0.05). However, post-operative incontinence was more frequent with PT (p = 0.027).

**Conclusion:**

PT can be an acceptable substitute of LAR in low income settings despite having higher incidence of incontinence.

## Highlights

• Low anterior resection (LAR) is the preferred surgery for rectal cancers.
• Coloanal pull-through (PT) used to be preferred before LAR.
• PT has higher complications rates, but it is much cheaper.
• PT might have similar survival outcomes compared to LAR.

## 1 Introduction

Colorectal cancers are the second most common cancers overall and are the third deadliest cancers. Many risk factors were identified such as family history, particular genes, history of smoking or radiotherapy, particular medical conditions such as diabetes, age, gender, BMI, diet and the environment. Rectal cancers are usually asymptomatic in early stages, but their symptoms range from rectal bleeding, and tenesmus to inability to completely evacuate the stool in addition to rectal and pelvic pain [1,2].

Treatment is indicated according to appropriate staging. Surgery can be curative and complete resection is the treatment of choice for rectal cancers. Neoadjuvant including chemoradiotherapy (CRT) is strongly recommended in stage 2 and 3 cancers (where the size is either T3, or T4 with or without nodules) and it does not increase surgical complications according to many clinical trials [2].

Complete resection is achieved with having negative proximal margins of 5 cm and distal marginas of 2 cm with having a radial greater than 1 mm. Surgery also aims to restore the continuity of the bowels. Surgeries through the abdomen are the preferred methods and there are mainly two types of surgeries according to sparing the sphincter depending on the location of the cancer and the distance between it and the sphincter; the first one is low anterior resection (LAR) which preserves the sphincter and the second one does not and is called abdominal perineal resection (APR). Using any of these approaches also depends on the staging and the ability to achieve the distal margins. Both procedures require total mesorectal excision (TME) to ensure having negative margins and lymph nodes retrieval. LAR is considered to be the gold standard technique [2]. Unfortunately, APR continues to be the treatment of choice for many cases where sparing the sphincter cannot be achieved [3]. For stages 2 and 3 cancers, CRT is recommended for all patients regardless of the surgery outcome [2].

Historically, coloanal pull-through (PT) was the first surgical procedure used to manage coloanal cancers in the mid-20th century, but now it is not commonly used as the surgery is much simplified by using staplers. Unfortunately, there are not many recent studies on this technique. However, PT is now having a comeback in cases with deferred anastomosis as it allows and protects it when there is a high risk of dehiscence because it avoids having a temporary faecal diversion [4].

Using coloanal anastomosis techniques such as LAR can have complications of dehiscence and pelvic abscess which can be life threatening and even in moderate cases, can substantially increase the duration of stay and cost [4]. One large study in Beijing, China found that LAR inpatients median cost was estimated to be around ￥89 064 [5] (which is around 13 758.25 USD).

Syria has been facing war for many years which caused more than 80 % of its population to be under poverty line. This caused the medical sector to suffer as many hospitals were destroyed, many doctors immigrated and the funding of the medical sector drastically dropped [6]. The public medical sector provides services for free. However, as the funding is low, many materials were not available which made it hard to conduct many procedures.

This study aims to evaluate PT and LAR procedures which were conducted in Damascus in a low-income setting to treat rectal cancers and to evaluate postoperative recurrence and complications.

## 2 Methods

This is a retrospective study from patients' records. Patients were admitted in Al-Assad University Hospital and Al-Mouwasat University Hospital where they had the surgery from August 2016 until December 2017. These two hospitals are considered among the major public hospitals across Syria as people from all Syria visit them to have surgeries. They provide free services to their patients, are located in the capital city of Damascus and were not directly damaged by war. Data was collected from the hospitals records after taking patients’ consents.

All patients were treated by the same group of surgeons. All patients received neoadjuvant therapy composed of (CRT) with a total irradiation dose of 45 Gy (25 × 1.8) and capecitabine (Xeloda®) 825 mg/m2, after surgery in cases of T3 and T4 or confirmed lymph node metastases post-operative adjuvant chemotherapy is required, FOLFOX which is made up of the drugs Folinic acind (leucovorin) “FOL”, Fluorouracil (5-FU) “F” and Oxaliplatin (Eloxatin®) “OX” or capecitabine (Xeloda®) 1225 mg/m2 is added. The previous protocols are the one used across Syria. Patients conduct either LAR or PT, depending on the availability of staplers in the hospital, not on the patient condition, which means that they were randomly chosen to conduct each surgery type.

Inclusion criteria in this study included having a tumour ranged from 4 to 12 cm from the anal verge, having a good prognosis of longer than 6 months, having a good sphincter function before the operation and not having other major comorbidities (uncontrolled diabetes for instance). Tumour size had to be smaller than 3.5 cm, not infiltrating the external anal sphincter and not having any evidence of the lymph nodes being affected before the surgery. Diagnosis made by biopsy and imaging by CT-scan to estimate the staging. Each case in PT group was matched by one from LAR group by tumour staging and prognosis. We chose 30 patients from each patient group who fulfilled the inclusion criteria for each surgery type. This number was chosen as it is adequate for the study purposes according to the ethical committee and hospitals policy as they are very busy and relying on paper-based documents, not electronic. We could not cross other variables then cancer type, its prognosis and staging.

Patient follow-ups in this study was for 1 year due to overflow in the hospital and patients not coming after this period. It was done by examination, imaging and colonoscopy and admission depending on the case and time after the surgery. Recurrences were managed by doing abdominal perineal resection in patients who had PT surgery. However, recurrence in LAR cases could not be surgically managed.

Ethical approval was taken from Damascus University ethical committee. Informed consent was also taken for collecting and publishing the data. This study had no funding.

This work has been reported in line with the Strengthening the reporting of cohort studies in surgery (STROCSS) criteria [7].

This research is registered under the unique identifying number of researchregistry6953.

### 2.1 Surgical technique

A diet on only liquids was implemented for at least one day before the surgery and as a hospital protocol, we used laxatives such as polyethylene glycol although they are not recommended in other hospitals. Furthermore, we used preoperative prophylaxes antibiotics and two surgical approaches were used: either LAR or PT. LAR required the use of stapler and cutting a section from the bower then conducting anastomosis and colostomy. In both procedures, negative margins were identified after resection according to the guidelines (5 cm proximal and 2 cm distal). TME was performed in both surgeries. Transabdominal open surgeries were the method used for both surgeries as well.

The surgical drain was installed and removed in day 5 in uncomplicated cases. Urinary catheters were used in both groups, and they were mostly removed on day 3 after the surgery. Faecal incontinence was evaluated 6 months after the surgery. The ability to have an erection was evaluated after 2 months of the surgery and after colostomy closure to reduce its effect on mentality. This was only evaluated in men who declared that they could have an erection before the operation.

Data was processed using IBM SPSS software version 25 for Windows (SPSS Inc, IL, USA). Chi-square, independent *t*-test and Fisher's exact were used and p values of less than 0.05 were considered significant.

## 3 Results

There were no operative or follow-up mortality (as we only followed-up for 1 year). Mean age was 49 years for LAR and 50 years for PT (Table 1). Post-surgical leak occurred after a mean number of days of 7 days for LAR (min: 5, max: 9), and 4 days for PT (min: 3, max: 5). The mean time to do LAR surgery was 160 min compared to 113 min for PT surgery (Table 1). When using independent *t*-test, LAR had significantly longer time to be conducted compared to PT (p < 0.001).

**Table 1:** Showing the gender, mean age and time spent in operation room for each surgery.

Characteristic	LAR (n = 30)	PT (n = 30)	Total (*n* = 60)
Gender **Male** **Female**	16 (53.3 %) 14 (46.7 %)	14 (46.7 %) 16 (53.3 %)	30 30
**Mean Age in years (** ± **SD)**	47.6 (±12.5)	49.2 (±12.7)	48.3 (±12.5)
**Mean time required of surgery in minutes (** ± **SD)**	161.0 (24.9)	106.3 (±19.3)	133.7 (±35.3)

LAR: Low anterior resection; PR: Pull-through.

Gender and complications are demonstrated in (Table 2). Around 50–100 ml was observed from the drain daily and it was mostly serous fluids without noticing any significant differences between the groups. After urinary catheter removal, three patients (2 LAR, 1 PT) experienced urinary retention and was managed by keeping the urinary catheter for a few months, and two patients had symptoms resolved in 3 months. However, one patient who conducted LAR procedure was still suffering from the retention for 9 months until the time of this study.

**Table 2:** Showing the gender and complications of patients who conducted either LAR or PT surgeries.

Characteristic	LAR (n = 30)	PT (n = 30)	Total (*n* = 60)	p value
Gender **Male** **Female**	16 (53.3 %) 14 (46.7 %)	14 (46.7 %) 16 (53.3 %)	30 30	0.606
Post-operative Leak **No leak** **Leak from the wound** **From the vagina** **From the surgical drain**	27 (90 %) 1 (3.3 %) 1 (3.3 %) 1 (3.3 %)	27 (90 %) 1 (3.3 %) 0 (0 %) 2 (6.7 %)	54 2 1 3	0.721
Post-operative urinary retention* No urinary retention **Positive urinary retention**	28 (93.3 %) 2 (6.7 %)	29 (96.7 %) 1 (3.3 %)	57 3	1.000
Post-operative stricture **No stricture** **Positive stricture**	28 (93.3 %) 2 (6.7 %)	25 (23.3 %) 5 (16.7 %)	53 7	0.254
Post-operative incontinence^#^ **Satisfactory function** **Partial incontinence** **Complete incontinence**	28 (93.3 %) 1 (3.3 %) 1 (3.3 %)	20 (66.6 %) 7 (23 %) 3 (10 %)	48 8 4	0.027
Sexual function^Ʈ^ **Able to have an erection** **Unable to have an erection**	11 (91.7 %) 1 (8.3 %)	12 (100 %) 0 (0 %)	23 1	0.590
Recurrence^α^ **No recurrence** **Positive recurrence**	28 (93.3 %) 2 (6.7 %)	28 (93.3 %) 2 (6.7 %)	56 4	1.000

LAR: Low anterior resection; PR: Pull-through.

Fisher's Exact and Chi square Tests were used.

* Urinary retention was observed after urinary catheter removal.

# Incontinence was evaluated 6 months after the surgery and partial incontinence means that there is incontinence for gases and/or loose stools.

Ʈ: Sexual function was evaluated by being able to have an erection after surgery in men who were able to have an erection before surgery.

α: Recurrence was evaluated by follow-ups for at least one year.

Two LAR patients suffered from strictures in the location of anastomosis, which was observed during the closure of the ileostomy, and five PT patients suffered from strictures and this was around 21 days after the surgery. Strictures were managed by dilation under general anaesthesia using Hegar dilators. This was successful in 6 patients, but one PT patient was still suffering from stricture until the time of this study (7 months after the procedure).

Sexual function was only studied in men who were able to have an erection before surgery, and it has been evaluated two months after surgery and after closing the ileostomy in order to isolate psychological factors. Two PT's males and four LAR's males were not able to have an erection before surgery. Only one LAR male patient was unable to have an erection after surgery.

Complete faecal incontinence (for gases and solids) was observed in one patient who had LAR surgery and in three patients who had PT surgery. However, only one LAR patient had partial faecal incontinence (for solid stool but not for gases and loose stools), whereas seven PT patients suffered from partial faecal incontinence. Recurrence was observed in two patients in each group.

Overall, we estimated that the cost of using LAR was at least 3 times higher than PT as using the cheapest stapler would cause an increase of at least 1000$ which is a large amount money for Syria.

## 4 Discussion

This study found that both procedures when clinically indicated can have similar outcomes and recurrence rate for the tumour. Post-operative stricture and incontinence were more prevalent in patients who conducted PT.

It was reported that strictures in the anastomosis were more common after PT and they were associated with adjuvant CRT. It was also found that there was no significant difference between the two procedures when using laparoscopy in the occurrence of post-operative incontinence [8].

One recent retrospective study on PT found similar complications of faecal incontinence and urinary and sexual functions when compared to manually fashioned coloanal anastomosis, even in cases with leaks and pelvic abscesses [4,9]. PT can also have a high rate of failure (25 %), but this can be attributed to the indication and severity of the case rather the type of PT used [4].

Another study reported that there was 3 % operative mortality and 36 % morbidity with PT. Furthermore, 10 % among the 36 % had a leak in the anastomosis, fistulae, and a pelvic sepsis. Around 14 % required a re-intervention and only 40 % reported having good or satisfactory functional results after one year and it reached 73 % after 2 years [10]. That study suggested that PT followed by TME and a delayed colo-anal anastomosis (DCA) could be safe and efficient to treat patients with middle or low rectal cancers and it allowed to preserve the sphincter and avoid a prophylactic diverting stoma [10].

### 4.1 Limitations

Sample size was not calculated before doing the study. We did not evaluate these procedures in high-grade cancers without being able to evaluate them in more complicated ones. Also, we did not identify risk factors in our patients, and we could not include patients with other comorbidities due to difficulty of evaluation and relatively small sample size. Finally, we could not perform longer follow-ups.

## 5 Conclusions

This study suggests that PT can provide an acceptable cost-effective replacement of LAR where there is no sufficient fund, time, or inpatient places. PT and LAR had similar tumour outcomes in our study. However, PT had higher post-operative incontinence and stricture rates. More studies are required with longer follow-ups, especially in more complicated cases to assess PT and whether certain adjustments can be implemented to improve the outcomes.

## Funding

We received no funding in any form.

## Please state any conflicts of interest

None to declare.

## Please state any sources of funding for your research

We received no funding.

## Ethical approval

We received Damascus University, faculty of medicine ethical approval for this study.

## Consent

Patients written consent was taken.

## Author contribution

### Authors' contributions

• AMG: Conceptualization; Data curation; Formal analysis; Project administration; Writing - review & editing; Resources.
• AK: Conceptualization; Supervision; Data curation; Formal analysis; Methodology; Validation; original draft; Writing - review & editing.
• AYG: Software; Resources; Conceptualization
• FOA**:** Investigation; Project administration; Methodology; Resources; Methodology; Writing - review.

## Registration of research studies

researchregistry6953

https://www.researchregistry.com/browse-the-registry#home/registrationdetails/60e642210ffe5700216fc55f/

## Guarantor

Fadi Obaid Alahmar is the guarantor.

## Declaration of competing interest

We have no conflict of interest to declare.
